# An Ideal Health Resort

**Published:** 1906-07-28

**Authors:** Conrad W. Thies


### AN IDEAL HEALTH RESORT.

In these days many persons prefer to spend their holidays abroad, and the numbers of English tourists who visit Switzerland and other popular Continental resorts increase each year. It is estimated that Lucerne alone now receives annually about one quarter of a million visitors, the larger proportion of whom are from England.

There is a strong tendency for English tourists to adhere to the beaten tracks, rather than venture to explore less well-known districts, with the inevitable result that the more popular resorts, such as Lucerne and the Bernese Oberland, are much overcrowded during the summer holiday season, while many beautiful and equally accessible districts are almost entirely neglected by English visitors.

It may interest some of your readers to hear of an ideal health resort in the Southern Austrian Tyrol which I have recently visited, after an illness, with most excellent results in the way of renewed health and vigour. This district is much frequented by both German and Italian visitors, who have for many years past appreciated its many attractions, but it is almost unknown to English travellers, although it is easily reached by various routes *via* Innsbruck and the Brenna Pass to the historic town of Trient, whence a branch railway ascends amongst the mountains to Roncegno, a village in the beautiful Val Sugani, through which runs the rapid River Brenta.

The Grand Hôtel des Bains at Roncegno is situated on a high plateau, about 2,000 feet above the sea-level, and the terrace in front of the hotel commands most extensive views of the charming valley, with its green meadows diversified by fine chestnut, laurel, and acacia trees, while on either side rise a series of high mountains. Those with a southern aspect have their slopes cultivated with vineyards and mulberry plantations almost to their summits, while the rugged peaks facing the north are capped with snow until quite late in the summer months.

The hotel provides accommodation for over 200 guests, and all its appointments are quite up to the high standard of the best modern hotels. The food is of excellent quality, and is cooked and served in the best manner; indeed, it would be difficult to find anywhere a better managed or more perfectly equipped establishment. A high-class stringed band discourses good music on the terrace or in the concert hall after both lunch and dinner.

The proprietor, Mr. Waiz, and most of the staff speak English, and do all in their power to add to the comfort of their English guests.

The bathing establishment, which is connected with the hotel, is replete with every modern appliance for bathing and every description of hydropathic treatment.

The mineral waters of Roncegno are well known as a most powerful agent for the cure of anæmia, catarrhal and skin affections, and many forms of nervous complaints.

The hotel is surrounded by an extensive park of about 50 acres, charmingly laid out with shady walks amidst a diversity of fine trees, amongst which are many pines. Numerous châlets and seats are provided, also lawns for tennis, so that visitors can obtain ample exercise and occupation without leaving the grounds. It would, indeed, be difficult to find a place more perfectly adapted for rest and recuperation.

For those, however, who are good walkers the immediate neighbourhood offers many attractions. Numerous interesting excursions can be made up or down the valley, while for the more robust there are ample opportunities for mountain climbing.

From the summit of several of the neighbouring mountains, which can be reached in from three to ten hours' excursions, the most extensive views can be obtained of the whole southern region of the Tyrol, including the Dolomites, while southwards can be seen the plains of Northern Italy and the Venetian Lagunes. The crests of the mountains on the south side of the valley are the border line between Austria and Italy. The Val Sugana is Austrian, but the people and their towns—Pergine, Levico, and Borgo—are Italian in character, and they present many novel features and picturesque sights to English visitors.

Conrad W. Thies.

